# Supplementary material for: A chromatogram-simplified Streptomyces albus host for heterologous production of natural products
Source: Antonie Van Leeuwenhoek. 2019 Nov 28;113(4):511–20. doi: 10.1007/s10482-019-01360-x (PMC7089911; doi:10.1007/s10482-019-01360-x)
Supplement: Supplementary file 1 — Supplementary file1 (DOCX 2680 kb) [file 10482_2019_1360_MOESM1_ESM.docx]

***Antonie van Leeuwenhoek Journal of Microbiology***

**Supporting Information**

**A chromatogram-simplified *Streptomyces albus* host for heterologous production of natural products**

Asif Fazal^1,2,3†^, Divya Thankachan^1,3†^, Ellie Harris^1,3^, Ryan F. Seipke^1,3^*

^1^School of Molecular and Cellular Biology, ^2^School of Chemistry, ^3^Astbury Centre for Structural Molecular Biology, University of Leeds, Leeds, LS2 9JT, United Kingdom

^†^Authors contributed equally to this work

*Correspondence:

E-mail: [r.seipke@leeds.ac.uk](mailto:r.seipke@leeds.ac.uk)

**Table of Contents**

Supporting figures and tables

**Figure S1.** Illumina read mapping to the antimycin BGC in *S. albus* S4 ∆5.

**Figure S2.** Illumina read mapping to the candicidin BGC in *S. albus* S4 ∆5.

**Figure S3.** Illumina read mapping to the albaflavenone BGC in *S. albus* S4 ∆5.

**Figure S4.** Illumina read mapping to the surugamide BGC in *S. albus* S4 ∆5.

**Figure S5.** Illumina read mapping to the fredericamycin BGC in *S. albus* S4 ∆5.

**Figure S6.** Full scale HPLC trace for *S. albus* S4 WT and *S. albus* S4 ∆5.

**Table S1.** Bacterial strains, cosmids and plasmids.

**Table S2.** Oligonucleotide primers used in this study.

**Figure S1.** Deletion of the entire antimycin BGC in *Streptomyces albus* S4 ∆5. Black rectangles represent Illumina MiSeq reads mapped to the *S. albus* S4 ∆5 genome. The red rectangle indicates the antimycin BGC.

**Figure S2.** Mutagenesis of the candicidin BGC in *Streptomyces albus* S4 ∆5. Black rectangles represent Illumina MiSeq reads mapped to the *S. albus* S4 ∆5 genome. Red rectangles indicate the homology-directed repair arms used to delete the *STRS402234* gene (also known as *fscC*). HDR, homology-directed repair arm.

**Figure S3.** Mutagenesis of the surugamide BGC in *Streptomyces albus* S4 ∆5. Black rectangles represent Illumina MiSeq reads mapped to the *S. albus* S4 ∆5 genome. Red rectangles indicate the homology-directed repair arms used to repair the double strand break generated by the Cas9 targeting the *STRS406449* gene. HDR, homology-directed repair arm; sgRNA, single guide RNA.

**Figure S4.** Mutagenesis of the albaflavenone BGC in *Streptomyces albus S4* ∆5. Black rectangles represent Illumina MiSeq reads mapped to the *S. albus* S4 ∆5 genome. Red rectangles indicate the homology-directed repair arms used to repair the double strand break generated by the Cas9 targeting the *STRS404430* gene. HDR, homology-directed repair arm; sgRNA, single guide RNA.

**Figure S5**. Mutagenesis of the fredericamycin BGC in *Streptomyces albus* S4 ∆5. Black rectangles represent Illumina MiSeq reads mapped to the *S. albus* S4 ∆5 genome. Red rectangles indicate the homology-directed repair arms used to repair the double strand break generated by the Cas9 targeting the *STRS406091* gene. HDR, homology-directed repair arm; sgRNA, single guide RNA.

**Figure S6**. Full scale version of the HPLC chromatogram depicted in Figure 3 in the main text.

**Table S1.** Bacterial strains, cosmids and plasmids used in this study

| **Strain/cosmid/plasmid** | **Description ^a^** | **Reference** |
| --- | --- | --- |
| *Candida albicans* | *Candida albicans* strain CA-6 | (Valiante et al. 2015) |
| *Micrococcus luteus* | *Micrococcus luteus* NCTC 9379 | NCTC |
| ***Streptomyces*** |  |  |
| S4 | Wild type *Streptomyces albus* S4 | (Barke et al. 2010) |
| ∆1 | S4 harbouring a complete deletion of the antimycin BGC | This study |
| ∆2 | ∆1 harbouring a deletion in the *STRS402234* (*fscC*) of the candicidin BGC | This study |
| ∆3 | ∆2 harbouring a deletion in the *STRS406449* gene of the surugamide BGC | This study |
| ∆4 | ∆3 harbouring a deletion in the *STRS404430* gene of the albaflavenone BGC | This study |
| ∆5 | ∆4 harbouring a deletion in the *STRS406091* gene of the fredericamycin BGC | This study |
| ∆5/Act | *S. albus* S4 ∆5 harbouring the actinorhodin BGC (*attB* ΦC31::pAH77); Apr^R^ | This study |
| ∆5/Cin | *S. albus* S4 ∆5 harbouring the cinnamycin BGC (*attB* ΦC31:: pIJ10109) ; Apr^R^ | This study |
| ∆5/Prun | *S. albus* S4 ∆5 harbouring the neoantimycin/prunustatin BGC split over two cosmids (*attB* ΦC31::cosmid813 *attB* ΦBT1::cosmid69); Apr^R^, Hyg^R^, Kan^R^ | This study |
| ***Escherichia coli*** |  |  |
| BW25113 | Host for REDIRECT PCR targeting system | (Gust et al. 2003) |
| NEBα | General cloning host | New England Biolabs |
| ET12567 | Non-methylating host for transfer of DNA into *Streptomyces* spp. (*dam, dcm, hsdM*); Cam^R^ | (MacNeil et al. 1992) |
| GB05-red | Host for RecET recombination | (Fu et al. 2012) |
| **Cosmids and BACs** |  |  |
| Supercos1 | Cosmid backbone for *S. albus* S4 Cosmid 213; Carb^R^, Kan^R^ | Stratagene |
| Cosmid 213 | Supercos1 derivative containing the entire antimycin biosynthetic gene cluster; Carb^R^, Kan^R^ | (Seipke et al. 2014) |
| Cosmid 213∆*antF* | Cosmid 213 derivative harbouring an apramycin-marked deletion of *antF*; Carb^R^, Kan^R,^ Apr^R^ | This study |
| Cosmid 213∆*antA*-*antO* | Cosmid 213 derivative harbouring an apramycin-marked deletion in the entire antimycin biosynthetic gene cluster (*antABCDEFGHIJKLMNO*); *antF*; Carb^R^, Kan^R,^ Apr^R^ | This study |
| Cosmid 213∆antA-antO-FLP | Cosmid 213∆*antA*-∆*antO* derivative in which the apramycin resistance cassette was removed by the FLP recombinase; Carb^R^, Kan^R^ | This study |
| Cosmid 213∆*antA*-*antO*-FLPHygoriT | Cosmid 213∆*antA*-*antO*-FLP derivative in which the *bla* resistance gene on the cosmid backbone was disrupted with a *hyg^R^-oriT* cassette | This study |
| pIJ10109 | Derivative of pOJ436 harbouring the cinnamycin biosynthetic gene cluster from *Streptomyces cinnamoneus*; Carb^R^, Apr^R^ | (Widdick et al. 2003) |
| Cosmid 813 | Supercos1 derivative harbouring *natABCDEF* from the neoantimycin biosynthetic gene cluster; Carb^R^, Kan^R^ | (Skyrud et al. 2018) |
| Cosmid 69 | Supercos1 derivative harbouring a partial *natB* gene and *natCDEFGQF’G’HIJKLNOP* genes from the neoantimycin biosynthetic gene cluster; Carb^R^, Kan^R^ | (Skyrud et al. 2018) |
| Cosmid 69- ΦBT1 | Cosmid 69 derivative engineered to integrate into the ΦBT1 *attB* site; Carb^R^, Hyg^R^ | (Skyrud et al. 2018) |
| **Plasmids** |  |  |
| pCRISPomyces-2 | pGM1190 derivative harbouring the CRISPR/Cas9 machinery; Apr^R^ | (Cobb et al. 2015) |
| pCRISPomyces-2-sur | Derivative of pCRISPomyces-2 derivative containing the *STRS406449*-targeting protospacer cloned into the BbsI site and homology-directed repair arms cloned into the XbaI site; Apr^R^ | This study |
| pCRISPomyces-2-alb | Derivative of pCRISPomyces-2 derivative containing the *STRS404430*-targeting protospacer cloned into the BbsI site and homology-directed repair arms cloned into the XbaI site; Apr^R^ | This study |
| pCRISPomyces-2-fdm | Derivative of pCRISPomyces-2 derivative containing the *STRS406091*-targeting protospacer cloned into the BbsI site and homology-directed repair arms cloned into the XbaI site; Apr^R^ | This study |
| pIJ773 | ReDirect PCR template plasmid harbouring an apramycin resistance cassette and *oriT*; Carb^R^, Apr^R^ | (Gust et al. 2003) |
| pIJ10701 | ReDirect PCR template plasmid harbouring a hygromycin resistance cassette and *oriT*; Hyg^R^ | (Gust et al. 2003) |
| pKC1132-UpDn | Derivative of suicide plasmid pKC1132 (Bierman et al. 1992)  containing ~3kb of homologous DNA upstream and downstream of the region of *fscC* targeted for deletion; Apr^R^ | (Seipke et al. 2011) |
| pUZ8002 | Encodes conjugation machinery for mobilisation of plasmids from *E. coli* to *Streptomyces;* Kan^R^ | (MacNeil et al. 1992) |

^a^ Carb, carbenicillin; Apr, apramycin; Hyg, hygromycin, Kan, kanamycin; Cam, chloramphenicol; *oriT*, origin of conjugal transfer

**Table S2.** Oligonucleotide primers used in this study

| **Primer alias** | **Sequence (5'-3')^a^** | **Description** |
| --- | --- | --- |
| RFS196 | **ctcgtgtcgttctcaggtggagaggtgcctgcgcgctca**tgtaggctggagctgcttc | PCR: *antF* REDIRECT knockout cassette |
| RFS236 | cgcctacaacaccggtgagt | PCR: confirmation of ∆ant mutation |
| RFS237 | aggggacgatgttgacgacc | PCR: confirmation of ∆ant mutation |
| RFS197 | **gacggccccggcggccgggacggccggcggtgcctgatg**attccggggatccgtcgacc | PCR: *antF* REDIRECT knockout cassette |
| RFS219 | **cacgcgcccgcgtgtctcaccccgccatggtggccgtca**attccg gggatccgtcgacc | PCR: *antABCDEHIJKLMNO* REDIRECT knockout cassette |
| RFS203 | **ccgcctcggccgggtcgggagacatctggcgggcggtca**tgtaggctggagctgcttc | PCR: *antABCDEHIJKLMNO* REDIRECT knockout cassette |
| RFS242 | atcacgcggctgatcgacca | PCR: confirmation of ∆*antF* mutant strain |
| RFS243 | tggaggaactgcggaccatc | PCR: confirmation of ∆*antF* mutant strain |
| EH_S3 | acgctgccgggccgccgcgagaaa | CRISPR protospacer targeting *STRS4_04430* (albaflavenone BGC) |
| EH_S4 | aaactttctcgcggcggcccggca | CRISPR protospacer targeting *STRS4_04430* (albaflavenone BGC) |
| EH_S7 | acgcgtacgcctgctccatggaga | CRISPR protospacer targeting *STRS4_06091* (fredericamycin BGC) |
| EH_S8 | aaactctccatggagcaggcgtac | CRISPR protospacer targeting *STRS4_06091* (fredericamycin BGC) |
| EH_S9 | acgccacctcacgcggcaccggga | CRISPR protospacer targeting *STRS4_06449* (surugamide BGC) |
| EH_S10 | aaactcccggtgccgcgtgaggtg | CRISPR protospacer targeting *STRS4_06449* (surugamide BGC) |
| EH_P7 | **tgccgccgggcgttttttat**gtgtactggttccgctc | PCR: *STRS4_04430* homology-directed repair arm |
| EH_P8 | **tttgttcgtgcctgctttcc**cgaatccaccaccgaac | PCR: *STRS4_04430* homology-directed repair arm |
| EH_P9 | **ggcgttcggtggtggattcg**ggaaagcaggcacgaac | PCR: *STRS4_04430* homology-directed repair arm |
| EH_P10 | **cggcctttttacggttcctggcct**ggttggatgcgaagagg | PCR: *STRS4_04430* homology-directed repair arm |
| EH_P15 | **tgccgccgggcgttttttat**cgctcttcctgctgttgttgg | PCR: *STRS4_06091* homology-directed repair arm |
| EH_P16 | **ggtcatgtggtagccgttgg**ttggcccttcacgatcatctcc | PCR: *STRS4_06091* homology-directed repair arm |
| EH_P17 | **agatgatcgtgaagggccaa**ccaacggctaccacatgacc | PCR: *STRS4_06091* homology-directed repair arm |
| EH_P18 | **cggcctttttacggttcctggcc**tatggtcttgaggtcgttgaagc | PCR: *STRS4_06091* homology-directed repair arm |
| EH_P19 | **tgccgccgggcgttttttat**gtacgtcatgtccacctcc | PCR: *STRS4_06449* homology-directed repair arm |
| EH_P31 | **cgtaagcctgggtgttgtcc**tacagctcgctcagttcg | PCR: *STRS4_06449* homology-directed repair arm |
| EH_P27 | **cgcgaactgagcgagctgta**ggacaacacccaggcttacg | PCR: *STRS4_06449* homology-directed repair arm |
| EH_P28 | **cggcctttttacggttcctggcct**ctccttcaccgacttcagc | PCR: *STRS4_06449* homology-directed repair arm |
| EH_P35 | gtcgtgaatctcctgatcg | PCR: confirmation of *STRS4_04430* mutation |
| EH_P36 | tacggctacctctacatcgacc | PCR: confirmation of *STRS4_04430* mutation |
| EH_P37 | tggccgaacccttctactcc | PCR: confirmation of *STRS4_06091* mutation |
| EH_P38 | ccatgtccaggtcgttcagc | PCR: confirmation of *STRS4_06091* mutation |
| EH_P39 | caccaggacttcttcacg | PCR: confirmation of *STRS4_06449* mutation |
| EH_P40 | gagggagaagaagttgtcgtgg | PCR: confirmation of *STRS4_06449* mutation |

^a^ non-homologous sequences are underlined and engineered restriction endonuclease sites are bolded

**References**

Barke J, Seipke RF, Grüschow S, Heavens D, Drou N, Bibb MJ, Goss RJM, Yu DW, Hutchings MI (2010) A mixed community of actinomycetes produce multiple antibiotics for the fungus farming ant *Acromyrmex octospinosus.* BMC Biol 8:109. doi: 10.1186/1741-7007-8-109

Bierman M, Logan R, O'Brien K, Seno ET, Rao RN, Schoner BE (1992) Plasmid cloning vectors for the conjugal transfer of DNA from *Escherichia coli* to *Streptomyces* spp. Gene 116:43–49. doi: 10.1016/0378-1119(92)90627-2

Cobb RE, Wang Y, Zhao H (2015) High-efficiency multiplex genome editing of *Streptomyces* species using an engineered CRISPR/Cas system. ACS Synth Biol 4:723–728. doi: 10.1021/sb500351f

Fu J, Bian X, Hu S, Wang H, Huang F, Seibert PM, Plaza A, Xia L, Müller R, Stewart AF, Zhang Y (2012) Full-length RecE enhances linear-linear homologous recombination and facilitates direct cloning for bioprospecting. Nat Biotechnol 30:440–446. doi: 10.1038/nbt.2183

Gust B, Challis GL, Fowler K, Kieser T, Chater KF (2003) PCR-targeted Streptomyces gene replacement identifies a protein domain needed for biosynthesis of the sesquiterpene soil odor geosmin. Proc Natl Acad Sci USA 100:1541–1546. doi: 10.1073/pnas.0337542100

MacNeil DJ, Gewain KM, Ruby CL, Dezeny G, Gibbons PH, MacNeil T (1992) Analysis of *Streptomyces avermitilis* genes required for avermectin biosynthesis utilizing a novel integration vector. Gene 111:61–68. doi: 10.1016/0378-1119(92)90603-M

Seipke RF, Barke J, Brearley C, Hill L, Yu DW, Goss RJM, Hutchings MI (2011) A single *Streptomyces* symbiont makes multiple antifungals to support the fungus farming Ant *Acromyrmex octospinosus.* PLoS ONE 6:e22028–8. doi: 10.1371/journal.pone.0022028

Seipke RF, Patrick E, Hutchings MI (2014) Regulation of antimycin biosynthesis by the orphan ECF RNA polymerase sigma factor σ^AntA^. PeerJ 2:e253. doi: 10.7717/peerj.253

Skyrud W, Liu J, Thankachan D, Cabrera M, Seipke RF, Zhang W (2018) Biosynthesis of the 15-membered ring depsipeptide neoantimycin. ACS Chem Biol 13:1398–1406. doi: 10.1021/acschembio.8b00298

Valiante V, Monteiro MC, Martín J, Altwasser R, Aouad El N, González I, Kniemeyer O, Mellado E, Palomo S, de Pedro N, Pérez-Victoria I, Tormo JR, Vicente F, Reyes F, Genilloud O, Brakhage AA (2015) Hitting the caspofungin salvage pathway of human-pathogenic fungi with the novel lasso peptide humidimycin (MDN-0010). Antimicrob Agents and Chemother 59:5145–5153. doi: 10.1128/AAC.00683-15

Widdick DA, Dodd HM, Barraille P, White J, Stein TH, Chater KF, Gasson MJ, Bibb MJ (2003) Cloning and engineering of the cinnamycin biosynthetic gene cluster from *Streptomyces cinnamoneus cinnamoneus* DSM 40005. Proc Natl Acad Sci USA 100:4316–4321. doi: 10.1073/pnas.0230516100
